# Antioxidative and Anti-Inflammatory Effects of *Lactobacillus plantarum* ZS62 on Alcohol-Induced Subacute Hepatic Damage

**DOI:** 10.1155/2021/7337988

**Published:** 2021-12-06

**Authors:** Yi Gan, Xiufeng Chen, Ruokun Yi, Xin Zhao

**Affiliations:** ^1^Chongqing Collaborative Innovation Center for Functional Food, Chongqing Engineering Research Center of Functional Food, Chongqing Engineering Laboratory for Research and Development of Functional Food, Chongqing University of Education, Chongqing 400067, China; ^2^Gastrointestinal Cancer Center, Chongqing University Cancer Hospital, Chongqing 400030, China

## Abstract

*Lactobacillus plantarum* ZS62 is a newly isolated strain from naturally fermented yogurt that might offer some beneficial effects in the setting of alcohol-induced subacute liver injury. The liver-protective effect of *L. plantarum* ZS62 was investigated by gavage feeding of mice with this *Lactobacillus* strain (1 × 10^9^ CFU/kg _BW_) before alcohol administration daily for 7 days. We then compared hepatic morphology, liver function indexes, liver lipid levels, inflammation, oxidative stress levels, and mRNA expression of oxidative metabolism- and inflammation-related genes in mice that had been pretreated with *Lactobacillus plantarum* versus control mice that had not been pretreated. Our results showed that *L. plantarum* ZS62 attenuated alcohol-induced weight loss; prevented morphological changes in hepatocytes; reduced markers of liver damage including aspartate aminotransaminase (AST), alanine aminotransaminase (ALT), hyaluronidase (HAase), precollagen III (PC III), and inflammatory cytokines; and enhanced the antioxidative status. *L. plantarum* ZS62 also significantly downregulated inflammation-related genes and upregulated lipid- and oxidative-metabolism genes. Thus, *Lactobacillus plantarum* pretreatment appears to confer hepatic protection by reducing inflammation and enhancing antioxidative capacity. The protective effect of *L. plantarum* ZS62 was even better than that of a commonly used commercial lactic acid bacteria (*Lactobacillus delbrueckii* subsp. *Bulgaricus*). The *L. plantarum* ZS62 might be a potentially beneficial prophylactic treatment for people who frequently drink alcoholic beverages.

## 1. Introduction

*Lactobacillus plantarum* is one of the lactic acid bacteria (LAB) that can be easily isolated from plant-based foods and naturally fermented foods. It has been widely used in the food industry, as well as in the manufacturing of health products. The strains used in functional foods usually have health-promoting characteristics, such as *Lactobacillus plantarum* P-8 [1], *L. plantarum* FLPLo5 [2], and *L. plantarum* LP3 [3]. These three strains are classified as probiotics which can be beneficial when consumed in adequate amounts (FAO/WHO). More and more strains have been shown to possess benefits in models of cardiovascular disease [4, 5], cancer [6], and liver disease [7, 8].

The liver is the primary organ of alcohol metabolism that is easily damaged by chronic and excessive alcohol consumption. ALD is a wide spectrum of liver lesions, ranging from steatosis to fibrosis/cirrhosis. ALD is a global healthcare problem that might be one of the oldest diseases of human beings due to alcoholic beverages that existed as early as circa 10,000 B.C [9]. In 2016, alcohol resulted in 5.3% of all deaths (some 3 million) worldwide. In China, 6% of males and 1% of females die from alcohol-related diseases [10]. Moreover, 51.1 males and 27.1 females per 100,000 population, respectively, have liver cirrhosis [11]. Simple alcoholic fatty liver is usually self-limited, and asymptomatic and can be recovered by abstinence. There are 5% to 15% patients, however, who still progress to fibrosis and cirrhosis after abstinence [12]. The development/progression of ALD is affected by dose, drinking patterns, duration, and so forth, among which the total amount of ingested alcohol is the most important factor [13]. Another important factor, binge drinking pattern, raises the risk of ALD, as well as the all-cause mortality [14]. Enzymes involved in the metabolism of alcohol, including alcohol dehydrogenase and acetaldehyde dehydrogenase, are also associated with the development of ALD.

The pathogenesis of ALD is not fully understood. Much evidence has suggested that probiotics may have the ability to prevent or treat ALD without side effects [15]. For example, *Lactobacillus rhamnosus* CCFM1107 [16], *L. fermentum* LA12 [17], and *L. plantarum* LC27 [18] can attenuate alcohol-induced liver injury. Furthermore, even heat-killed strains and breaking solutions can ameliorate ALD [19, 20]. Numerous studies have reported that probiotics can attenuate alcoholic liver damaged mainly via improving lipid metabolism [21], reducing TNF-*α* [22], IL-6 [23], and IL-1*β* [24], and ameliorating oxidative stress [25]. Reducing oxidative stress includes reducing MDA levels, increasing SOD activity [23], and regulating the keep-Nrf2-ARE signaling pathway [20].

In this study, we investigated if *Lactobacillus plantarum* ZS62 has antioxidative and anti-inflammation effects in a model of alcohol-induced hepatic injury in mice. Regulatory effects on the related gene expression were explored to further elucidate the mechanism of hepatic protection conferred by this bacterial strain. This avenue of investigation may help to develop beneficial probiotic products for consumption by humans.

## 2. Materials and Methods

### 2.1. Strains

*L. plantarum* ZS62 was isolated from naturally fermented yogurt in Zhaosu, Xinjiang, China, identified in NCBI based on the Basic Local Alignment Search Tool (BLAST), and preserved in the China General Microbiological Culture Collection Center (CGMCC, No. 18228). A commonly used commercial strain, *Lactobacillus delbrueckii* subsp. *bulgaricus* (CGMCC No. 1.16075), was purchased as a comparison. Both strains were stored at -80°C and activated in MRS liquid medium for 24 h before use; the cultures were centrifuged (10 min, 3000 rpm) to collect cells and resuspended in normal saline.

### 2.2. Mice Models of Alcohol-Induced Subacute Hepatic Damage

Male Kunming mice [26] (forty, 6-week-old), purchased from Experimental Animal Center of Chongqing Medical University (Chongqing, China), were acclimated to the appropriate environment (25 ± 2°C, relative humidity 55 ± 5%, and 12-h day/night cycle), with food and water provided ad libitum. After 7 days' adaptation, mice were randomly grouped (*n* = 10) into the control group (control), model group (model), alcohol and *Lactobacillus delbrueckii* subsp. *bulgaricus* fed group (alcohol+LDSB), and alcohol and *L. plantarum* ZS62 fed group (alcohol+ZS62).

**Figure 1:**
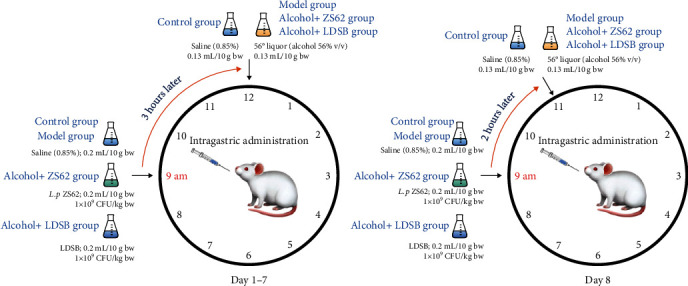
Treatments during the experiment. Control: mice without treatments except twice 0.85% saline treatment a day; model: mice treated with alcohol (56%, v/v, 0.13 mL/10 g _bw_); alcohol+LDSB: mice treated with of 1.0 × 10^9^ CFU/kg_bw_*Lactobacillus delbruechill* subsp. *Bulgaricus* before alcohol treatment; alcohol+ZS62: mice treated with 1.0 × 10^9^ CFU/kg_bw_*Lactobacillus plantrum* ZS62 before alcohol treatment.

During the experiment, all groups received normal diets, twice daily gavage administration, and daily weightings. At 9 : 00 am, mice in the control and model group received gavage administration of 0.85% (v/v) saline solution, and alcohol+LDSB and alcohol+ZS62 received gavage administration of LDSB and *L. plantarum* ZS62, respectively; 3 hours later (12 : 00 am), mice in the control group also received gavage administration of 0.85% (v/v) saline solution, and mice in the model, alcohol+LDSB and alcohol+ZS62, received gavage administration of alcohol (Figure 1). The 56° liquor (56% alcoholic beverage, v/v) was given at 0.13 mL/10 g BW and provided 5.82 g_alcohol_/kg_bw_. On the eighth day, all the groups were euthanized (fasted for 12 h) before the treatment in Figure 1 (the first gavage administrations were also performed at 9 : 00 am, and the second gavage administrations were performed and 11 : 00 am). The viscera (heart, kidney, and liver) were obtained to calculate the organ indexes (viscus weight/body weight). Blood was collected and centrifuged (4°C, 3500 rpm, 10 min) for serum preparation. The liver was homogenized (10% homogenate) and centrifuged (4°C, 4000 rpm, 10 min) to obtain the supernatant.

### 2.3. Morphological Observation

Liver samples were fixed in formalin (10%, v/v), dehydrated, embedded in paraffin, and sliced. Then, the morphologies of samples were observed after been stained by hematoxylin and eosin (H&E) method.

### 2.4. Detections of Serum Indexes

The enzyme activities of AST, ALT, HAase, and PC III and contents of interleukin- (IL-) 10, IL-1*β*, IL-6, and tumor necrosis factor-*α* (TNF-*α*) in serum were measured using purchased kits (Nanjing Jiancheng Bioengineering Institute, Nanjing, Jiangsu, China).

### 2.5. Measurement of Liver Indexes

The liver levels of total cholesterol (TC), triglyceride (TG), enzyme activities of alcohol dehydrogenase (ADH) and acetaldehyde dehydrogenase (ALDH), and oxidative stress-related indexes of malondialdehyde (MDA), superoxide dismutase (SOD), and glutathione (GSH) were detected by purchased kits (Nanjing Jiancheng Bioengineering Institute, Nanjing, Jiangsu, China).

### 2.6. Measurement of the mRNA Expression

The hepatic total RNA was extracted by the TRIzolTM Reagent method [27]. Relative mRNA expression of C-Jun N-terminal kinase (JNK), extracellular regulated protein kinases (ERK), cyclooxygenase-1 (COX1), peroxisome proliferator-activated receptor-*α* (PPAR-*α*), nuclear factor erythroid-2 related factor 2 (Nrf2), heme oxygenase-1 (HO-1), SOD1, SOD2, glutathione peroxidase (GSH-Px), catalase (CAT), and nicotinamide adenine dinucleotide phosphate (NADPH) were detected by quantitative real-time reverse transcription-polymerase chain reaction (RT-PCR). Glyceraldehyde-3-phosphate dehydrogenase (GAPDH) also was detected as an internal reference. The purity and concentration of RNA were determined, adjusted to 1 *μ*g/*μ*L, and used as a template in reverse transcription PCR (20 *μ*L reaction system). The relative gene expression was calculated according to the 2^−*ΔΔ*Ct^ method [27].

### 2.7. Statistical Analysis

The determinations were performed in triplicate and presented as the mean ± standard deviation. The data were analyzed (SPSS Inc., Chicago, IL, USA), and statistical significance (*p* < 0.05) was determined by a one-way analysis of variance using the Duncan multirange test.

## 3. Results

### 3.1. Body Weight Variation

**Figure 2:**
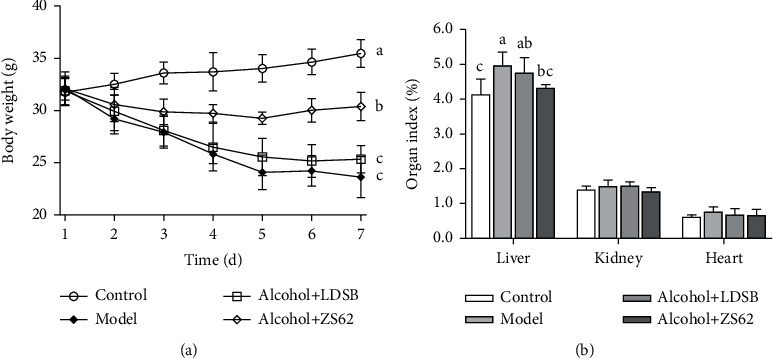
Changes of body weight (a) and organ indexes (b) of each group. The different alphabets (^a–c^) mean significant differences (*p* < 0.05). Control: mice without treatments except twice 0.85% saline treatment a day; model: mice treated with alcohol (56%, v/v, 0.13 mL/10 g _bw_); alcohol+LDSB: mice treated with of 1.0 × 10^9^ CFU/kg_bw_*Lactobacillus delbruechill* subsp. *Bulgaricus* before alcohol treatment; alcohol+ZS62: mice treated with 1.0 × 10^9^ CFU/kg_bw_*Lactobacillus plantrum* ZS62 before alcohol treatment.

During the experiment, the bodyweight of the control group increased from 31.75 ± 1.29 g to 35.47 ± 1.34 g (Figure 2(a)), while that of the model significantly decreased from 32.13 ± 1.59 g to 23.62 ± 1.09 g (*p* < 0.05). At the same time, alcohol+LDSB and alcohol+ZS62 also decreased 25.33 ± 1.09 g and 30.38 ± 1.37 g, respectively. However, the magnitude of weight loss was decreased by treatment with *L. plantarum* ZS62 that significantly lighter than those of model and alcohol+LDSB (*p* < 0.05).

### 3.2. Organ Indexes of the Mice

The organ indexes are shown in Figure 2(b). The liver proportion to body weight of control (4.14 ± 0.43%) was significantly lower than that of model (4.96 ± 0.38%; *p* < 0.05). The liver index of alcohol+ZS62 (4.32 ± 0.09%) was significantly lower than that of model (*p* < 0.05), while that of alcohol+LDSB (4.75 ± 0.44%) was at the level of model (*p* > 0.05). The kidney and heart index of each group, however, was the same (*p* > 0.05).

### 3.3. Liver Pathological Observation

**Figure 3:**
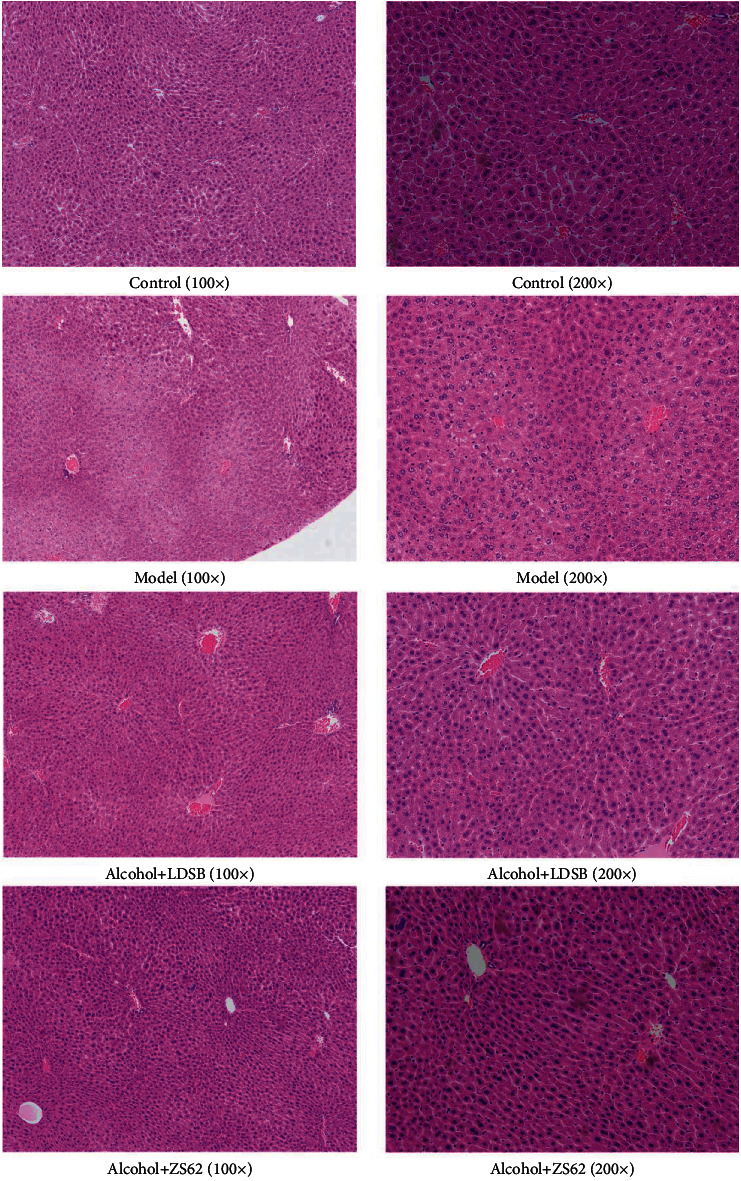
Hematoxylin-eocin (H&E) staining results of liver in mice. Magnification ×100 and ×200. Control: mice without treatments except twice 0.85% saline treatment a day; model: mice treated with alcohol (56%, v/v, 0.13 mL/10 g _bw_); alcohol+LDSB: mice treated with of 1.0 × 10^9^ CFU/kg_bw_*Lactobacillus delbruechill* subsp. *Bulgaricus* before alcohol treatment; alcohol+ZS62: mice treated with 1.0 × 10^9^ CFU/kg_bw_*Lactobacillus plantrum* ZS62 before alcohol treatment.

The stained liver tissue slices (×100, ×200) were shown in Figure 3. Hepatic morphology of the control group exhibited normal organized liver structures and lobular architecture, centered nuclei, and uniform cell size, while the structures of mice from the model group were disorganized, with no clearly defined boundaries nor centered nuclei. Moreover, significant hepatic inflammation was observed in the model group. The alcohol+LDSB showed a similar morphology to the model, but the inflammation stratus was alleviated to some degree. At the same time, the morphologies of alcohol+ZS62 were similar to those of control that demonstrated organized structures and clearly defined boundaries.

### 3.4. Serum Indexes of Each Group

**Figure 4:**
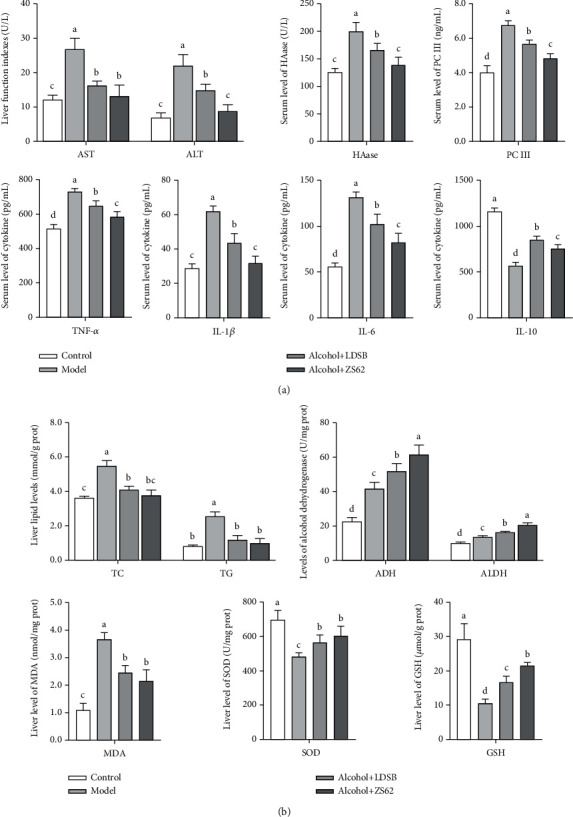
Serum and liver indexes of mice in each group: (a) Serum levels of ALT, AST, HAase, PC III, TNF-*α*, IL-6, IL-1*β*, and IL-10. (b) Liver levels of TC, TG, ADH, ALDH, MDA, SOD, and GSH. The different alphabets (^a–d^) mean significant differences (*p* < 0.05). Control: mice without treatments except twice 0.85% saline treatment a day; model: mice treated with alcohol (56%, v/v, 0.13 mL/10 g _bw_); alcohol+LDSB: mice treated with of 1.0 × 10^9^ CFU/kg_bw_*Lactobacillus delbruechill* subsp. *Bulgaricus* before alcohol treatment; alcohol+ZS62: mice treated with 1.0 × 10^9^ CFU/kg_bw_*Lactobacillus plantrum* ZS62 before alcohol treatment.

The serum indexes of AST, ALT, HAase, PC III, IL-6, IL-1*β*, and TNF-*α* in the Model group were markedly elevated relative to the control group and IL-10 which is an anti-inflammatory cytokine was noticeably lower than those of control (*p* < 0.05; Figure 4(a)). Both the LAB interventions blocked this alcohol-induced increase in the activities of AST, ALT, and HAase, and concentration of PC III and proinflammatory factor (IL-6, IL-1*β*, and TNF-*α*) and significantly increased the concentration of IL-10 (*p* < 0.05). The levels of AST, HAase, and IL-1*β* of alcohol+ZS62 dropped to the same levels of control (*p* > 0.05). The concentration of IL-10 of alcohol+LDSB was significantly higher than that of alcohol+ZS62 (*p* < 0.05).

### 3.5. Liver Indexes of Each Group

The hepatic concentration of TC and TG was significantly increased by alcoholic damage (*p* < 0.05). This rise was blocked by treatment with LDSB and *L. plantarum* ZS62 (*p* < 0.05; Figure 4(b)). Moreover, the level of TG in both LAB intervention groups was reduced to the same level of control (*p* > 0.05). The activities of ADH and ALDH were increased by 7-day induction by alcohol (*p* < 0.05); the activities of both enzymes were further enhanced by treatment with LDSB and *L. plantarum* ZS62 (*p* < 0.05), and the activity of alcohol+ZS62 was noticeably higher than that of alcohol+LDSB (*p* < 0.05).

The activity of SOD and the concentration of GSH was severely lowered by alcohol which was 69.31% and 36.17% of control (697 ± 54 U/mg port, 29.15 ± 4.54 *μ*mol/mg protein) that consequently elevated the level of MDA in the liver (*p* < 0.05). Fortunately, the hepatic activities of SOD in alcohol+LDSB and Aacohol+ZS62 group were increased to 566 ± 43 U/mg prot and 603 ± 56 U/mg prot, respectively, so was the concentration of GSH that climbed to 16.69 ± 1.80 *μ*mol/mg prot and 21.54 ± 0.90 *μ*mol/mg prot (LDSB, ZS62, respectively). So, the concentration of MDA in the alcohol+LDSB and alcohol+ZS62 groups dropped significantly (*p* < 0.05).

### 3.6. Relative mRNA Expression in the Liver

**Figure 5:**
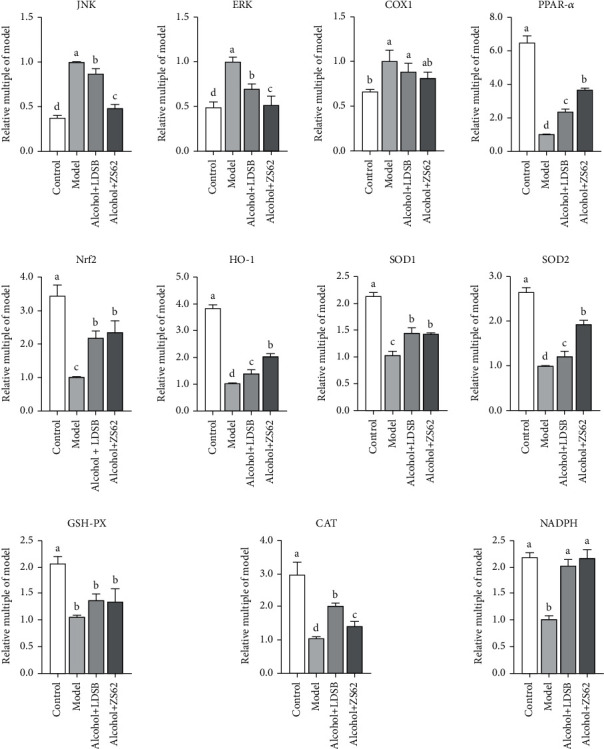
Hepatic mRNA expressions of mice; c-Jun N-terminal kinase (JNK), extracellular regulated protein kinases (ERK), cyclooxygenase-1 (COX1), peroxisome proliferator-activated receptor-*α* (PPAR-*α*), nuclear factor erythroid-2 related factor 2 (Nrf2), hemeoxygenase-1 (HO-1), superoxide dismutase 1 (SOD1), superoxide dismutase 2 (SOD2), GSH-Px (glutathione peroxidase), catalase (CAT), and nicotinamide adenine dinucleotide phosphate (NADPH) mRNA. The different alphabets (^a–d^) mean significant differences (*p* < 0.05). Control: mice without treatments except twice 0.85% saline treatment a day; model: mice treated with alcohol (56%, v/v, 0.13 mL/10 g _bw_); alcohol+LDSB: mice treated with of 1.0 × 10^9^ CFU/kg_bw_*Lactobacillus delbruechill* subsp. *Bulgaricus* before alcohol treatment; alcohol+ZS62: mice treated with 1.0 × 10^9^ CFU/kg_bw_*Lactobacillus plantrum* ZS62 before alcohol treatment.

Figure 5 illustrates the effects of alcohol on the expression of mRNA expression of *JNK*, *ERK*, and *COX1*, which were significantly downregulated (*p* < 0.05). The expression of *PPAR-α*, *Nrf2*, *HO-1*, *SOD1*, *SOD2*, *GSH-Px*, *CAT*, and *NADPH* were markedly upregulated by alcohol (*p* < 0.05) when compared to control. Meanwhile, gavage administration of *L. plantarum* ZS62 strikingly regulated all the genes above (*p* < 0.05) except *COX1* and *GSH-Px* (*p* > 0.05); the LDSB showed a similar but weaker effect on these genes. The expression of *NADPH* in both LAB groups was upregulated to the same level of control (*p* > 0.05). There were no significant differences in the expression of *COX1* between model and LAB groups; however, the *L. plantarum* ZS62 also helped to downregulate the expression to the control level (*p* > 0.05). Even the regulation effect of LDSB on *CAT* was greater than that of *L. plantarum* ZS62 (*p* < 0.05), the overall regulation effect of *L. plantarum* ZS62 was more pronounced than that of LDSB (*p* < 0.05).

## 4. Discussion

Alcoholic beverages include distilled spirits, fermented spirits, mixed spirits, and premixed spirits that contain 0.5% or higher alcohol content by volume. Alcoholic beverages have been consumed for social entertainment and leisure for thousands of years across the world. However, excess alcohol can lead to multiple behavioral and health problems. Despite the fact that alcohol provides 8.75 kcal/g, the weights of our alcohol-fed mice decreased. The degree of weight loss was closely connected with the drunk durations (time of losing righting reflex) which were 12 ± 1.4 h, 10 ± 1.6 h, and 5 ± 1.6 h for model, alcohol+LDSB, and alcohol+ZS62, respectively.

The liver is the most important organ for alcohol metabolism. During alcohol metabolism, the production of free radicals results in oxidative stress, as well as inflammation. This damages hepatocyte morphology and function, possibly leading to liver fibrosis [28]. Serum AST and ALT are medical indicators of liver function which significantly increase during liver dysfunction. In the progression of liver fibrosis, HAase and PC III are sensitive indexes that can accurately reflect the degree of liver damage. The increased concentrations of TC and TG in our model of alcohol-induced liver damage also suggested the aggravation of lipid accumulation and disorder of lipid metabolism [29]. The elevated levels of AST, ALT, HAase, and PC III suggest that the liver was injured by the alcohol consumption, and the rising levels of TC and TG show that lipid metabolism was disrupted (Figure 4). The decreased levels of these indexes in mice administered our putative therapeutic suggest that the liver dysfunction has been relieved. Thus, the LDSB and *L. plantarum* ZS62 alleviate the adverse effects of alcohol on liver functions.

The increased inflammatory cytokine levels reflect the level of severity of liver damage. Among these inflammatory cytokines, IL-6 is initially produced when inflammation occurs. IL-6 is a multifunctional cytokine of the acute phase response [30]. The increased serum IL-6 indicates that intake of alcohol causes an acute phase response (Figure 4(a)). Studies have described that increased serum IL-6 is associated with alcohol cravings and that IL-6 levels drop during abstinence [31]. TNF-*α*, a neurobiological marker, is related to alcohol consumption and takes part in the regulation of alcohol consumption [32]. However, the phenomenon of eagerness to drink was not observed in this study. Increased levels of IL-1*β*, a proinflammatory cytokine, also could aggravate alcoholic induced inflammation [33], while enhancing IL-10, an anti-inflammation cytokine important in immunomodulation after alcohol intake [34]. Our results showed similar results in that the proinflammatory cytokines were remarkably increased, and anti-inflammation cytokines were decreased by 7-day of induction, and the treatment with LDSB and *L. plantarum* ZS62 effectively reduced the production of proinflammatory agents and increased the anti-inflammation factor levels (Figure 4(a)). Even the concentration of IL-10 in alcohol+LDSB was significantly higher than that of alcohol+ZS62, the measured proinflammatory levels of alcohol+ZS62 were remarkably lower than those of alcohol+LDSB. And combined with Figure 3, *L. plantarum* ZS62 showed an excellent effect on alleviating inflammation status.

ADH and ALDH are important enzymes that participate in alcohol metabolism that converts alcohol to acetaldehyde by ADH and the following are oxidized to carboxylic acids by ALDH. Figure 4(b) demonstrates that alcohol-gavage increased the activates of both enzymes (model), and the *L. plantarum* ZS62 showed a further beneficial effect that helped to accelerate alcohol metabolism that can lessen the damage of acetaldehyde on the liver. These results might explain the attenuated inflammatory status of alcohol+ZS62.

Reactive oxygen species (ROS) are well known to cause oxidative stress when overproduced; the excess acetaldehyde also is converted to superoxide, consequently aggravating oxidative stress [35]. Scavengers of ROS and antioxidants, such as SOD and GSH, are important in reducing oxidative stress on the body. The lipid peroxidation end product, MDA, is also used as a marker of oxidative stress. Figure 4(b) shows that the levels of SOD and GSH in this model were sharply decreased, and the level of MDA was increased, which indicated that the body might be easily attacked by ROS and increased oxidative stress. Abnormal cellular morphology in Figure 3 was consistent with this assumption. At the same time, gavage administration of *L. plantarum* ZS62 significantly enhanced ROS scavenging capacity. The reduced MDA level was also associated with lower concentrations of hepatic TC and TG.

JNK is a subclass of the mitogen-activated protein kinase (MAPK) signaling pathway easily activated by ROS, TNF, and IL. This pathway can promote the occurrence and development of inflammation and the progression of liver fibrosis [36, 37]. ERK is another important member of the MAPK family activated in the metabolic syndrome that transfers massages into the nucleus and takes part in the processes of physiology and pathology [38]. Both JNK and ERK associate with collagen production and degradation and also could raise IL-6 levels [39, 40]. COX1, an isozyme of COXs, responds to inflammation and has become a target of inflammation treatment [41]. The upregulated expressions of *JNK* and *ERK* (Model) were significantly downregulated by the treatment of *L. plantarum* ZS62, and the expression of *COX1* was somewhat downregulated (Figure 5). Combined with the serum levels of IL-1*β*, IL-6, TNF-*α*, and IL-10 in Figure 4, *L. plantarum* ZS62 showed the ability to be anti-inflammatory.

PPAR-*α* is a ligand-induced nuclear receptor highly expressed in the liver, closely associated with biooxidation and fatty acid *β*-oxidation, and currently is used to treat dyslipidemia for it exerts an anti-inflammatory effect [42]. The relatively higher levels of TC and TG (Figure 4(b)) are associated with the lower expression of *PPAR-α* which reduced the speed of lipid metabolism and increased lipid accumulation. Both LAB strains upregulated the expression of *PPAR-α* and dropped the concentration of liver TC and TG.

Nrf2 is one of the antioxidant markers that can prevent hepatocytes from necroptosis and a transcription factor for the expression regulation of HO-1 which is another antioxidant marker [43, 44]. Activated Nrf2 is regarded as response protection against alcohol-induced liver damage. Alcohol downregulated the expression of both Nrf2 and HO-1 which indicated that the antioxidative capacity of the model was reduced; the intervene of *L. plantarum* ZS62 effectively upregulated the expression of *Nrf2* indicating that this strain responds to alcoholic oxidative stress.

SOD is the first detoxification enzyme of superoxide radical neutralization, among which SOD2 exists in the mitochondrial matrix and neutralizes superoxide radicals. SOD1, another species of SOD, converts harmful H_2_O_2_ produced by the dismutation reaction of SOD2 into O_2_ and H_2_ [45]. H_2_O_2_ also can be decomposed by CAT and GSH-Px into O_2_ and H_2_O [46]. Moreover, CAT reduces fat accumulation by degrading hepatic fatty acids without oxidative damage [47]. NADPH is a hydrogen carrier playing the role of reducing agent in biosynthesis and the coenzyme of GSH reductase by which the intracellular content can be kept [48].

The downregulated expression of *SOD1*, *SOD2*, *GSH-Px*, and *CAT* reduced the activities of antioxidant enzymes and consequently weakens the antioxidant capacity, and the downregulated expression of *NADPH* reduced the delivery of hydrogen and the content of GSH that also contributes to alcoholic oxidative stress. At the same time, both *L. plantarum* ZS62 and LDSB upregulated these genes that significantly enhanced the antioxidative ability of alcohol-fed mice, and the overall regulation of *L. plantarum* ZS62 on these genes was greater than that of LDSB. All in all, *L. plantarum* ZS62's hepatoprotective effect against alcoholic subacute liver damage might be attributable to its antioxidant and anti-inflammatory activities.

## 5. Conclusions

This study suggested that a dose of *L. plantarum* ZS62 at 1.0 × 10^9^ CFU/kg_BW_ could protect the liver from alcoholic damage by inhibiting alcohol-induced weight loss, alleviating inflammatory status and downregulating the expression of related genes, improving liver function, and upregulating the expression of antioxidant-related genes that enhance the antioxidant status. *L. plantarum* ZS62 probably resists alcohol-induced subacute liver damage via stimulating antioxidative and anti-inflammation pathways. This study suggests that *L. plantarum* ZS62 might be a potentially useful probiotic strain and that further studies on hepatic protection are warranted.

## Data Availability

The datasets generated for this study are available upon request to the corresponding author.
